# A Review on the Application of Silver Nanoparticles in Oral and Maxillofacial Surgery

**DOI:** 10.1055/s-0041-1731589

**Published:** 2021-08-24

**Authors:** Diane Isabel Selvido, Bishwa Prakash Bhattarai, Apiwat Riddhabhaya, Kadkao Vongsawan, Siripen Arunpraphan, Natthamet Wongsirichat

**Affiliations:** 1 International College of Dentistry, Walailak University, Bangkok, Thailand

**Keywords:** antibacterial resistance, implantology, oral surgery, silver nanoparticles, wound healing

## Abstract

Silver nanoparticles (AgNPs) have been taken advantage of in dentistry because of their good antibacterial resistance and self-sustaining potential. However, in oral and maxillofacial surgery and implantology, there is a lesser amount of evidence. The few pieces of evidence need to be accentuated for possible amplification of its use in the dental setting. AgNPs in oral and maxillofacial surgery can be used in wound healing, bone healing, extractions, guided tissue regeneration, apical surgeries, oral cancer, and dental implants. This review aims to feature the utilization and application of AgNPs in oral and maxillofacial surgery and implant dentistry, emphasizing its need for potential future development in clinical settings.

## Introduction

Controversy in the use of silver in health has been discussed over the years, especially for biological use. With the advent of many innovations, the use of silver in health care application has improved since the 1920s. Evidence has shown that silver particles tend to act profoundly on the bacteria it encounters, even at minimal concentrations. 1 Silver microfine particles at less than 20 nm in diameter, having increased solubility and ion-release, are recommended for wound healing. 2 Synthetization rendered silver particles as valuable and effective through the advancements in nanotechnology. 3

Silver nanoparticles (AgNPs) have been used in dentistry primarily because of their antimicrobial properties. 4 In prosthodontics, the acrylic resin applied with this material has shown promising results, as it impeded the growth of *Candida albicans* in its biofilm. 5 Tissue conditioners integrated with AgNPs also concluded bactericidal effects. 6 Versatility in endodontic use ranged from irrigants and medicaments to coating materials for gutta-percha. 7 8 9 10 Similarly, in restorative dentistry, light-cured composite resins have been modified with AgNPs and showed various findings, again highlighting their antimicrobial properties. 11 12 13 13

The activity of AgNP counts on many factors such as surface chemistry, morphology, particle size, cell type, among others. 14 AgNPs are usually 1 nm to 20 nm in size. 15 The particles commonly used in dentistry are less than 10 nm, maximizing their bactericidal and bacteriostatic properties. 16 Ion release and antimicrobial activity are more enhanced when finer AgNPs such as those 10 nm in size are utilized. 16 Because of their small dimensions, the surface area is taken advantage of, passing more into cell membranes, thereby contributing to augmented antimicrobial action. 17 18 The potency is said to be commensurate to the amount of ionic release of particles. 1 Silver nanoparticles have been shown to have this effect on pathological bacterial strains. 19 Principally, Zbuchea et al stated that the activity behind the antimicrobial property lies in the production of Ag ^+^ in an aqueous atmosphere. 20 Silver ions can also adhere and denature DNA and RNA, reducing bacterial progression. 21 However, certain reviews say that the mechanism behind the antimicrobial activity remains not fully understood. 19 22 Even so, knowledge of the AgNPs will help us maximize their full potential and for safe administration. Not only does it have the bacteria-resisting ability, but it also possesses antiviral and antifungal properties. 23 24

AgNPs do not come without shortcomings, as it is known to cause irritations. Thus, another preparation called colloidal silver is made to combat these adverse effects. 21 Colloidal silver is described as AgNPs stabilized in water and is considered to be an alternative medicine. 25 The indirect antibacterial action occurring at this condition neutralizes the enzymes responsible for bacterial replication. 26

Toxicity concerning AgNPs in laboratory studies has garnered different results, depending on particles' size, concentration, surface, and charge. 17 27 28 However, the smaller the nanoparticle size, the better penetration of the silver ions and the better the antibiotic effect. 29 Moreover, Talapko et al also mentioned that AgNP toxicity remains unclear. 30 Argyria, a mucosal staining condition affected by silver deposits, can occur in the dermis of the skin but was not emphasized in the oral cavity. In addition to that, silver uptake in the mouth was stated to be of minimal risk. 1 Reports have been made on wound dressing discolorations because of prolonged application but were yet reported in dental surgery treatments. 31

The use of AgNPs is prevalent in dentistry, as previously mentioned. However, concerning oral and maxillofacial surgery, its use is worth paying attention to, as it was not thoroughly investigated. This review aims to feature the utilization and application of AgNPs in oral and maxillofacial surgery and implant dentistry, emphasizing its need for potential future development in the clinical settings.

## Materials and Methods

This is a review about AgNPs and their other related forms in oral and maxillofacial surgery and dental implants. All searches were done through PubMed and other reputable sources on AgNPs precisely related to oral surgery. The journal articles and other related literature reviewed are associated with AgNPs in oral surgery procedures, either in vivo or in vitro, which will contribute to the field of interest.

## Synthesizing Silver Nanoparticles

Biologically adapted AgNPs are said to have properties known to produce increased solubility and stability. 32 According to Zhang et al, compared with synthetic methods in making AgNPs, the biological and “green” approaches are easy, fast, self-sustaining, and ideal. 4 33 34 In contrast, physical and chemical techniques in synthesizing AgNPs are deemed to be costly and unsafe. 32 35 Moreover, chemical means can deplete energy that leads to harmful effects in the environment. 36 37 Greener selections of AgNPs are integrated using antioxidants from various plants, different fruits, coffee, and tea extracts. 38

## Silver Nanoparticle Applications in Different Procedures

### Wound Healing

The oral and maxillofacial surgery and implant dentistry fields primarily deal with wounds most likely occurring within the oral cavity, either with incision, extraction, or trauma. Wound healing that it undergoes entails a step-by-step modulated process, defined as the destruction of the epithelium membrane, which may or may not require damage to the connective tissue and its structures underneath. The presence of cytokines, growth factors, and mediators helps achieve wound closure. 39 40 41 AgNPs contribute to wound healing by infiltrating into the Gram-positive's thick peptidoglycan layer and the Gram-negative's thinner layer containing lipopolysaccharides and acids. The more these particles get exposed, the more it becomes immersed to react into inflammation, proliferation, and tissue remodeling. 18 42 More specifically, wound healing in AgNPs was also supported by Tian et al, saying that AgNPs function by reducing the process of inflammation by controlling the inflammatory mediators’ behaviors. 43

Pharmaceutical manufacturers have developed silver-based wound dressings over time, depending on the type of wound, severity of infection, and clinical presentations but not particular to the dental field. They are claimed to be efficacious and are proven safe. 44 A recent study by Sugiharti et al in rats concluded that periodontal dressing mixed with AgNPs demonstrated prompt tissue recovery that may be suggestive of further clinical use. 45

### Simple Extractions

Few studies have demonstrated the efficacy of AgNPs and their derivatives. Cai and Lu inserted Gelatamp, a commercially available resorbable gelatin sponge containing colloidal silvers, into extraction sockets and concluded that it prevents the postoperative sequelae that can occur. The study described Gelatamp as a scaffold that continuously releases silver ions, reinforcing the antibacterial properties into the tissues untill 4 weeks after the intrasocket application. 46 Similarly, in Ragab and Melek's clinical trial in 2019 with patients undergoing anticoagulant therapy, Gelatamp was also used. The patients who used Gelatamp after tooth extraction achieved superior immediate postoperative hemostasis than the controls. 47 Likewise, the study by Piry et al determined that Gelatamp has the ability to promote hemostasis, with a significant reduction of pain symptoms between teeth with Gelatamp and control group. 48 This study interestingly implies that colloidal silvers in gelatin sponges are contributory to rapid hemostasis and wound healing, and might decrease the chances of other postoperative complications.

### Alveolar Osteitis

Alveolar osteitis is one of the complications of oral surgery. Nitzan et al have established in an in vitro study (1983) that *Treponema denticola* is one of the primary causative pathogens for it. Even so, the etiology remains unclear for this kind of infection. 49 For this purpose, the use of AgNPs and other derivatives to combat pathogenic bacteria became the interest of some studies aimed to prevent alveolar osteitis. An interesting finding by Maani et al used Gelatamp in the extraction sockets of patients who regularly receive anticoagulant therapy, warfarin, and those who discontinued it. Results have shown that Gelatamp is effective with or without warfarin, causing control of postoperative bleeding and proper healing of the extraction sockets, without signs of infection. 50 Supporting evidence was also revealed in a randomized clinical trial of Wang et al, where mandibular impacted third molar sockets applied with Gelatamp had minimal alveolar osteitis. Out of 1,350 surgically removed teeth, only 0.44% had the incidence of alveolar osteitis, 2% with gel foam, and 4.44% with the control group. 51 With consistent outcomes shown, it can be drawn that colloidal silver can be a promising agent in the deterrence of postoperative infections.

### Bone Regeneration

Intending to investigate the effect of gelatin sponges with colloidal silvers, Dong et al examined it involving Sprague–Dawley rats. The assessment was made by placing the scaffold with colloidal silver into the cranial defects. New bone formation was seen 2 to 4 weeks after, with compelling antimicrobial outcomes. In other words, the combination of gelatin and colloidal silver can promote bone formation in rat models. 52 However, in oral and maxillofacial surgery, the studies on bone healing properties of silver remain sparse.

As discussed by Sivollela et al in a review, antimicrobial membranes are innovated to lessen the chances of infections causing failure of graft placements in the mouth. 53 A novel ridge augmentation technique by Yang and Ouyang in 2015 used Gelatamp above the deproteinized bovine bone matrix after posterior extractions. Five teeth in four patients were recalled 4 to 6 weeks postoperatively, and it was reported that wound closure was achieved in the absence of infections in every case. Since the colloidal silver sponge was used, it was mentioned in their study that it provided protection by preventing leakage of the bone graft used, and it aided wound stabilization that is crucial for bone regenerating outcomes. 54 However, the role of AgNPs as an antimicrobial material was not featured in this investigation.

AgNPs were also developed in one experimental study by Lee et al in 2018, incorporating this substance into a chitosan membrane. They tested for the biocompatibility and effectiveness as a bacterial-resistant barrier, especially on *Porphyromonas gingivalis* . They analyzed that small concentrations of AgNPs can be used as a safe and reliable method when applied in guided tissue regeneration. 55 This evidence is congruent with Shao et al, where animal models are used. In addition to resistance to *Porphyromonas gingivalis* , *Fusobacterium nucleatum* was also indicated, thereby suggesting this blend as possible for dental practical application. 56

Platelet-rich fibrin has bone regenerating properties that are beneficial in ridge augmentation procedures. With that being said, Khorshidi et al in 2018 included AgNPs in leukocyte and platelet-rich fibrin (L-PRF) and showed compelling findings: the addition of AgNPs in L-PRF generated greater antimicrobial effects compared with L-PRF alone. Moreover, they concluded that this modification had improved the biomechanical properties of a membrane material needed for oral surgery use. 57

### Periapical Surgery

In an *in vivo* experiment on an animal model by Mena-Alvarez et al in 2019, researchers placed Gelatamp and other hemostatic agents. Periapical defects treated with Gelatamp indicated prolonged healing with an inflammatory reaction, citing the presence of polymorphonuclear cells at 6 weeks. 58 The findings contrast with the studies mentioned earlier that AgNPs and their other products promote wound healing.

### Dental Implant Surface Modifications

Since dental implants are retained in the cavity from its installation, there is a given susceptibility of disease inflicted by bacterial colonization. 59 60 To address the problems that come with dental implants, nano silvers are used in the modification of the surfaces to prevent biofilm growth. 61 Ordinarily, endosseous implant surface alterations are known to improve biomechanical properties by enhancing the surface area and wettability, promoting osseointegration and long-term stability. 62 Integrating AgNPs into titanium fixtures involves different processes that mainly include immersing the implant body into AgNO _3_ and different concentrations. 63

Flores et al analyzed in their study the antibacterial effect of AgNPs against *Pseudomonas aeruginosa* . These nanoparticles continuously adsorb on the titanium, producing nanometer-sized aggregates comprising AgNPs that surround the surface. The result indicated that a titanium implant body with AgNPs can produce excellent bacterial protection, highlighting its use in the dental setting. 64

Zhao et al, in their study, revealed that the AgNPs modified in titania nanotubes in titanium implants achieved antibacterial action against *Staphylococcus aureus* . Moreover, a definitive reduction of planktonic bacteria was observed. The bacterial resistance is enough for 30 days, which is crucial for primary stability. 63

In terms of the biocompatibility of dental implants, Lu et al measured the titanium implants integrated with different concentrations of AgNPs through cell cultures. The study suggests that for enhancing the biocompatible outcomes, heat treatments should be done. 65

An advanced method, by Zhong et al, of conditioning the titanium surface initially with phase-transited lysozyme and AgNP thereafter showed antimicrobial outcomes and soft-tissue healing. The priming of phase-transited lysozyme reinforced the antiinfection properties of the material. 66

AgNPs filled with nanoporous silica coatings were also done by Massa et al and effectively eliminated *Actinomyces actinomycetemcomitans* , a major periodontal pathogen that causes failure of implants. 67

Targeting *Lactobacillus salivarius* in the investigation by Boutinguiza et al with the incorporation of AgNPs, using laser ablation in an open atmosphere in dental implants, is also worth taking note of. 68 Coupled with the findings made by the in vitro studies mentioned on titanium implants, the findings by Boutinguiza et al support that coating dental implants with AgNPs exhibit resistance against bacterial strains. Furthermore, the mentioned ability of the AgNP in the fixture can be beneficial during the early days of dental implantation.

### Oral Cancer

According to Noronha et al, the innovation and development of AgNPs in oral cancer therapy can lead to novel strategies in the treatment of this disease. However, there are very scarce studies on this matter. 69 AgNPs have been agents of various studies that targeted various kinds of cancer cells but were recommended for a full understanding before clinical application. 70 Despite the effect of low-dose AgNP causing DNA fragmentation and condensation, it is essential to emphasize that there had been no harmful effect on normal cells. 71

Cell viability and proliferation of squamous cell carcinoma using AgNPs with or without alkaloid berberine were determined in a study by Dziedzic et al. It was found that a low concentration of AgNPs decreases cell viability and proliferation, implying that it is a potential cancer therapeutic agent. 72

Another study on cytotoxicity was given importance by Rosarin et al. They synthesized AgNP in *amla* plant extract using green methods. By testing the cytotoxicity, oxidative stress, and apoptosis in Hep 2 laryngeal carcinoma, it was found that AgNPs caused tumor-suppressing properties. 73

Strengthening the AgNP outcomes on oral cancer cytotoxicity, an in vitro experiment by Satapathy et al, utilizing a hybrid nanoparticle of bioactive quinacrine and silver, proved that the nanomaterial caused cytotoxicity and inhibited angiogenesis, which is a crucial feature of tumor progression in oral cancers. 74

## Limitations

There are limited studies with regard to AgNPs in oral and maxillofacial dentistry and implant dentistry procedures. Most of them are in vitro studies, which is much anticipated, as there is a necessity for greater evidence before proceeding with clinical treatments. Furthermore, the use of nanosilver and its many forms in oral and maxillofacial surgery and implant dentistry calls for more innovative methods to take advantage of its great affinity for preventing infections.

## Conclusion

Studies on AgNPs in the field of oral and maxillofacial surgery and implant dentistry have been expanding and developing. Many pioneering and feasible in vivo methods have been done, and these are somehow getting closer to pursuing safer methods on clinical use. Gelatamp, colloidal silver in a resorbable gelatin sponge that is commercially used, has been applied safely and efficaciously in a few studies, and future clinical research based on this material is encouraged for confirmation. The need for other AgNPs application, with dental surgery in mind, is recommended, highlighting the effect on wound dressings, suturing materials, and other surgical approaches. In addition, as AgNP toxicity remains unclear, the need for further investigation is also suggested in this issue.
